# miRNA-221 promotes cutaneous squamous cell carcinoma progression by targeting PTEN

**DOI:** 10.1186/s11658-018-0131-z

**Published:** 2019-03-08

**Authors:** Zhen-Hua Gong, Feng Zhou, Chao Shi, Tie Xiang, Chang-Kai Zhou, Qian-Qian Wang, Ya-Su Jiang, Sheng-Feng Gao

**Affiliations:** 1 Department of Burn and Plastic Surgery, The First People’s Hospital of Nantong, Nantong, 226001 China; 2 Department of Clinical Laboratory, The First People’s Hospital of Nantong, Nantong, 226001 China; 3 Department of Pathology, The First People’s Hospital of Nantong, Nantong, 226001 China

**Keywords:** Cutaneous squamous cell carcinoma, miRNA-221, PTEN, Proliferation, Treatment

## Abstract

**Background:**

Cutaneous squamous cell carcinoma (CSCC) is a common type of skin malignancy. MicroRNA-221 (miRNA-221) is a critical non-coding RNA in tumor initiation and progression. However, the molecular mechanisms of miRNA-221 in the development of CSCC remain unknown. This study investigated the expression of miRNA-221 in CSCC and its potential tumor biological functions.

**Methods:**

MTT assay, colony assay, PCR, and Western blot were adopted.

**Results:**

In this study, miRNA-221 expression was significantly higher in CSCC tissues and cell lines than in normal tissues and cells (*P* < 0.05). Further functional experiments indicated that miRNA-221 knockdown inhibited the proliferation and cell cycle, while upregulation of miRNA-221 presented the opposite role. The dual reporter gene assays indicated that PTEN is a direct target gene of miRNA-221. PTEN protein or mRNA levels were decreased after the cells were transfected with miR-221 mimics.

**Conclusions:**

Taken together, the obtained results indicated that miR-221 plays an oncogenic function in CSCC by targeting PTEN and further suggest that miR-221 may be a potential target for CSCC diagnosis and treatment.

## Background

Cutaneous squamous cell carcinoma (CSCC) is an epidermal keratinocyte derived skin tumor, which ranks as one of the most malignant cancers worldwide [1]. The primary risk factor for the occurrence of CSCC is UV exposure [2]. A large portion of patients have developed an aggressive form of CSCC by the time of diagnosis, which often metastasizes to other organs [3]. The long-term prognosis for these highly metastatic diseases is extremely poor, with a disease-specific survival at 1 year of 44–56% [4]. Thus, it is urgent to search for new diagnostic biomarkers and thereby improve the CSCC treatment outcome.

miRNAs are non-coding, single-stranded, small RNAs (22–24 nt) that can regulate gene expression by directly binding to the 3′-untranslated regions (3′-UTRs) of target messenger RNAs (mRNAs) [5, 6]. By regulating the protein expression of their target genes, miRNAs are implicated in a broad range of important cellular processes, including cell proliferation, apoptosis, migration, invasion and tumorigenesis [7–11]. miRNAs could act as novel oncogenes or tumor suppressors, depending on the roles of their target genes.

miR-221 is a member of the miR-221/222 cluster, which is located on the X chromosome. miR-221 has several conserved seed sequences which are identical to its homologous miRNA, miR-222 [12]. In particular, miR-221 expression level is reported to be up-regulated in several types of human cancers, including hepatocellular carcinoma [13], prostate cancer [14], and colon cancer [15], suggesting the oncogenic role of miR-221 in cancer initiation and progression. Conversely, in lung cancer miR-221 exhibits tumor suppressor roles [16]. The authors reported that miR-221 suppressed growth in four lung cell lines. The above results highlighted the dual functions of miR-221 in different cancer types. Therefore, it is necessary to explore the specific role of miR-221 in specific cancer types. The role of miR-221 in the progression of CSCC and its underlying mechanism, however, remains obscure.

In the current investigation, we found that miR-221 was significantly up-regulated in CSCC tissues and cell lines. In vitro experiments showed that silencing of miR-221 promoted CSCC cell growth. We also identified PTEN as a target of miR-221. Together our data suggest that miR-221 plays an oncogenic role in CSCC and further imply that miR-221 may be a novel target for diagnosis and treatment of CSCC in the near future.

## Methods

### Tissue samples

A total of 64 pairs of CSCC tissues and adjacent non-tumor tissues were collected from patients. All tissues were immediately stored in liquid nitrogen after surgery. This study was approved by the Medical Ethics Committee of The First People’s Hospital of Nantong. Written informed consent was collected from each patient.

### Cell culture

CSCC cell lines (SCC13, A431, HSC-5 and SCL-1) and human normal skin cell line (HaCaT) were bought from the Cell Bank of Type Culture Collection, Chinese Academy of Sciences (Shanghai, China). Cells were maintained in RPMI-1640 (GIBCO, US) along with 10% fetal bovine serum at 37 °C, in a humidified air with 5% CO_2_.

### Cell transfection

Cells (3 × 10^5^/per well) were seeded into 6-well plates for 12 h. 50 nM miR-221 mimics, miR-221 inhibitor or scrambled miRNA control (miR-NC) (GenePharma, Shanghai, China) were mixed with Lipofectamine 2000 reagent (Thermo Fisher Scientific, Inc.) for 15 min at room temperature in 200 μL of FBS-free medium. After that, the mixed medium was added into the well. After 48 h, total protein and RNA were extracted and then subjected to Western blotting and qRT-PCR analysis.

### RNA extraction and qRT-PCR

Total RNA was extracted from tissues and cell lines using the Trizol reagent (Invitrogen, Carlsbad, CA, USA). 1 μg of RNA was then reversely transcribed into cDNA using PrimeScript RT master Mix (Takara, Dalian, China). Then the PCR reaction was performed in the Applied Biosystems 7900 Fast Real-Time PCR system (Applied Biosystems, Foster City, California, USA) under the guidance of the manufacturer’s protocols. GAPDH and U6 were used for normalization for mRNA and miRNA, respectively. 2^–ΔΔCt^ method was adopted to calculate the relative gene expression.

### Cell proliferation assay

After transfection, cells (2 × 10^3^ cells/well) were seeded into 96-well plates and maintained at 37 °C in 100 μl of culture medium. After transfection for 24, 48, 72 and 96 h, MTT (5 mg/mL, 30 μL) was added to each well Then, after removing the medium, 100 μL of DMSO (Sigma) was added to solubilize the crystals and the absorbance was measured at 450 nm. Experiments were performed in triplicate and repeated at least three times independently.

### Colony formation assay

Cells were seeded at 500 cells in 6-well plates with 0.6% agarose underlay. After 14 days, colonies stained with crystal violet (Sigma-Aldrich) with more than 50 cells were counted.

### Cell cycle assay

1 × 10^6^ cells per well were seeded into the 6-well plates and incubated overnight. After transfection for 48 h, cells were harvested and washed gently with PBS. Cells were stained with propidium iodide (PI) and then were analyzed by the Modfit LT software (Verity Software House, US).

### Western blot

The proteins was extracted in RIPA buffer and then separated in 10% SDS-PAGE, and transferred onto PVDF membranes (Millipore, Billerica, MA, USA). Membranes were blocked with 5% (*v*/v) milk first and then incubated with primary antibodies overnight at 4 °C. After incubating with horseradish peroxidase (HRP)-conjugated anti-mouse antibody (1:2000) (DakoCytomation), protein blots were visualized by ECL (GE Healthcare). β-actin was used as a loading control.

### Luciferase report assay

The wild type (WT) and mutant type (MUT) of PTEN 3′-UTR luciferase reporter gene plasmids were generated by Yearthbio (Changsha, China). The cells were then co-transfected with miR-NC or miR-221 mimic, and with the WT or MUT of PTEN-3′-UTR reporter plasmid using Lipofectamine 2000 reagent. After incubation at 37 °C for 48 h, luciferase activities were detected using dual-luciferase activity assays (Promega, Madison, WI, USA).

### Statistical analysis

Data are presented as the mean ± SD (standard deviation). Statistical significance for differences between groups was determined by Student’s t-test or one-way ANOVA. *P* values of < 0.05 were considered significant.

## Results

### miR-221 level is increased in human CSCC tissues and cell lines

**Fig. 1:**
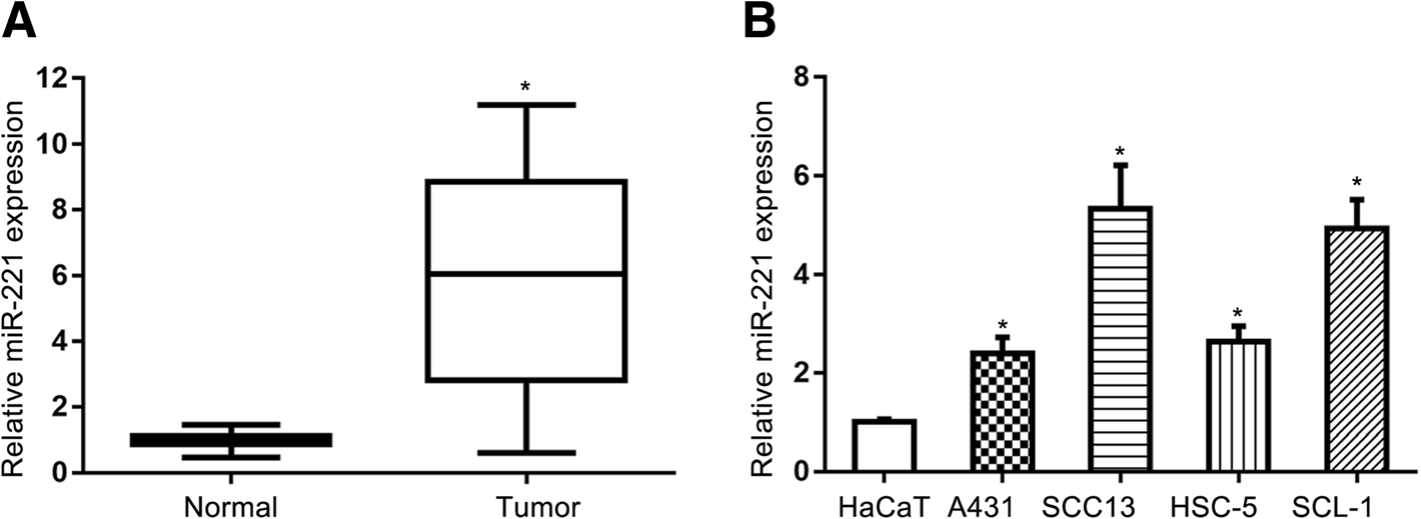
Expression of miR-221 in CSCC tissues and cell lines. **a** qPCR analysis of miR-221 in tumor and adjacent non-tumor tissues. **b** Average relative miR-221 level in CSCC cell lines (A431, SCC13, HSC-5 and SCL-1) and the human normal skin cell line HaCaT. Data are means ± SD of three independent experiments. * *P* < 0.05, compared with control

The miR-221 levels were determined by RT-qPCR in CSCC and adjacent non-tumor tissues. As shown in Fig. 1a, miR-221 expression was upregulated in the CSCC tissues compared with the adjacent noncancerous tissues. Consistently, all involved CSCC cell lines (SCC13, A431, HSC-5 and SCL-1) had significantly higher miR-221 levels than the human normal skin cell line HaCaT (Fig. 1b).

### miR-221 promotes growth of CSCC cells

**Fig. 2:**
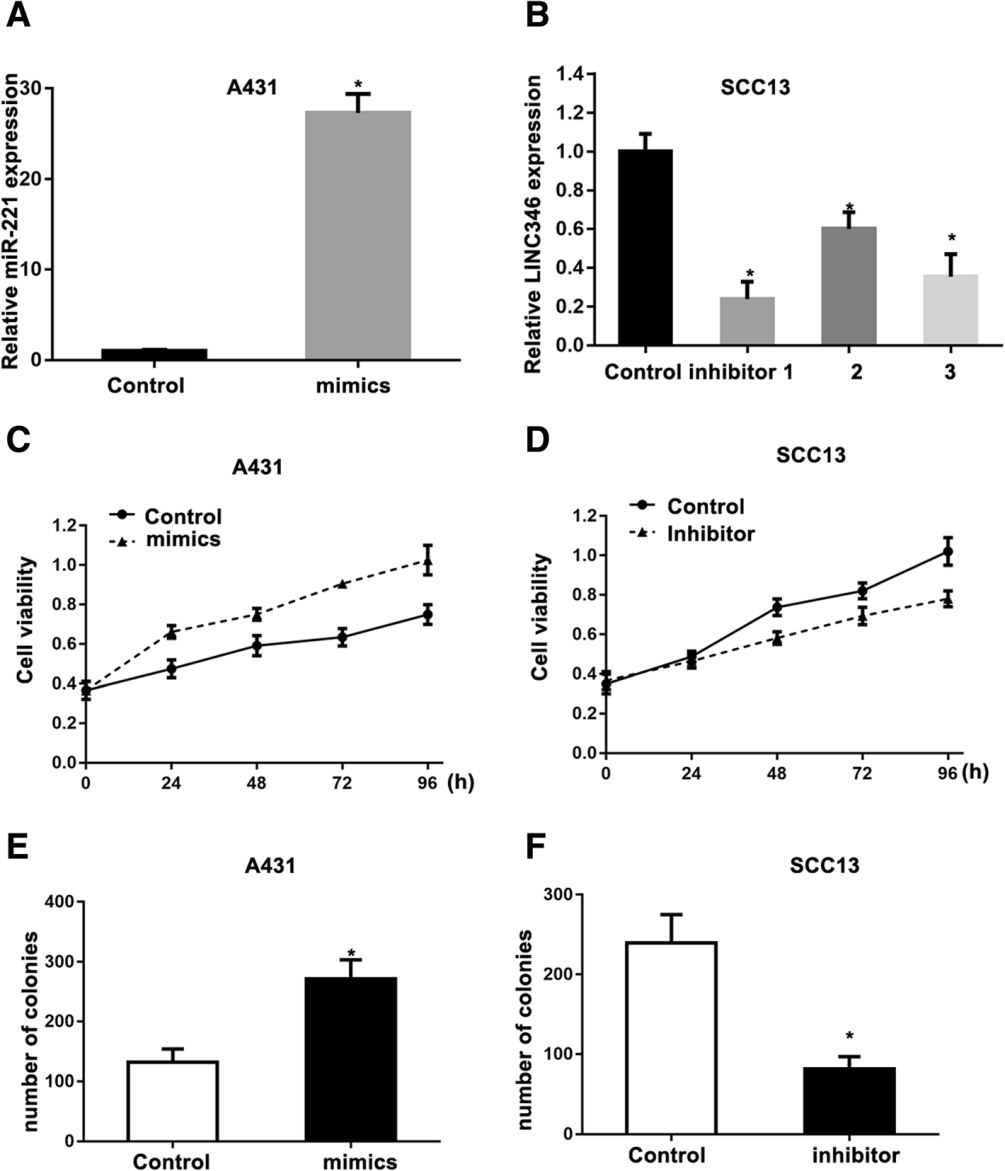
miR-221 regulates cell proliferation in CSCC. Expression level of miR-221 in cells after transfection with miR-221 mimics (**a**) and inhibitors (**b**), as detected by quantitative RT-PCR. miR-221 overexpression (**c**) or silencing (**d**) regulated cell viability in cells at 24, 48, and 72 h by MTT assay. Quantitative results of colony formation assay in A431 (**e**) and SCC13 (**f**) cells transfected with miR-221 inhibitor or mimic, respectively. * *P* < 0.05, compared with control

RT-qPCR was adopted to detect the level of miR-221 after miR-221 mimics or inhibitor treatment. The results showed that miR-221 mimics enhanced the expression of miR-221 in A431 cells (Fig. 2a), and miR-221 inhibitor dramatically downregulated the expression of miR-221 in SCC13 cells (Fig. 2b). To determine the exact functional roles of miR-221 in CSCC cell lines, we evaluated the cell proliferation by MTT after miR-221 mimics or inhibitor transfection. We observed that up-regulation of miR-221 significantly promoted cell proliferation (Fig. 2c), while down-regulated expression of miR-221 significantly inhibited cell proliferation (Fig. 2d). Moreover, the colony formation assay indicated that cells transfected with miR-221 mimic formed more colonies than cells transfected with control (Fig. 2e), and the opposite result was found in the cells transfected with miR-221 inhibitor (Fig. 2f).

### miR-221 promotes cell cycle of CSCC cells

**Fig. 3:**
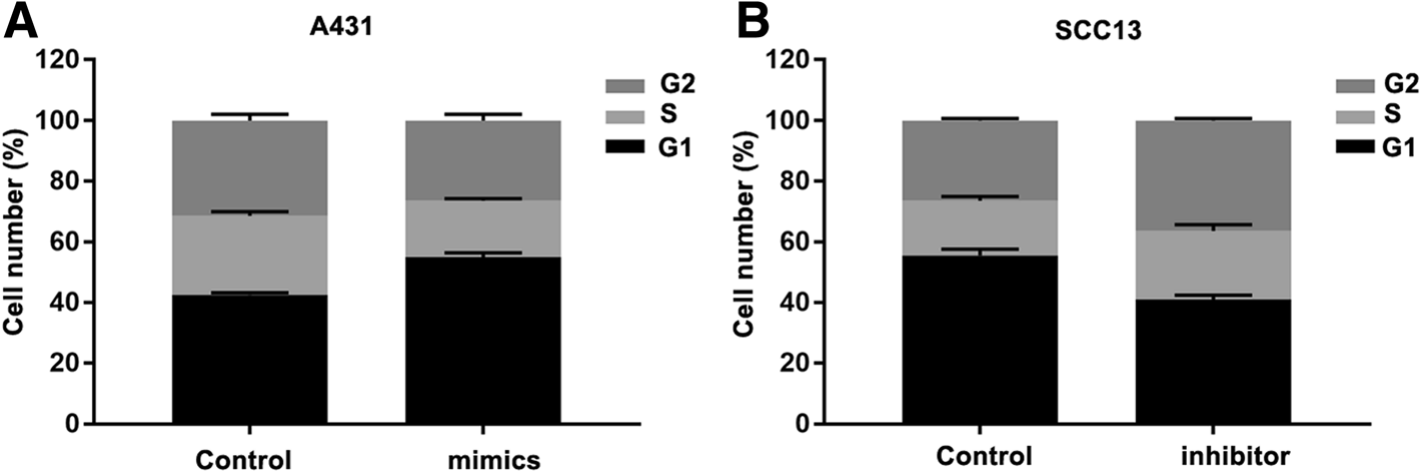
miR-221 regulates cell cycle in CSCC. Quantitative results of cell-cycle assay in A431 (**a**) and SCC13 (**b**) cells transfected with miR-221 inhibitor or mimic, respectively. * *P* < 0.05, compared with control

We further used flow cytometry assay to examine the impact of miR-221 in the cell cycle distribution. We observed that the G0/G1 phase fraction of the control group was less than that of the miR-221 mimic group, with 43.4 ± 5.8% compared to 67.5 ± 6.1% (Fig. 3a), whereas knockdown of miR-221 in cells had fewer cells in the G0/G1 phase, but more cells in the G2/M phase (Fig. 3b). These results revealed that miR-221 can promote the progression of the cell cycle.

### PTEN is a direct target of miR-221

**Fig. 4:**
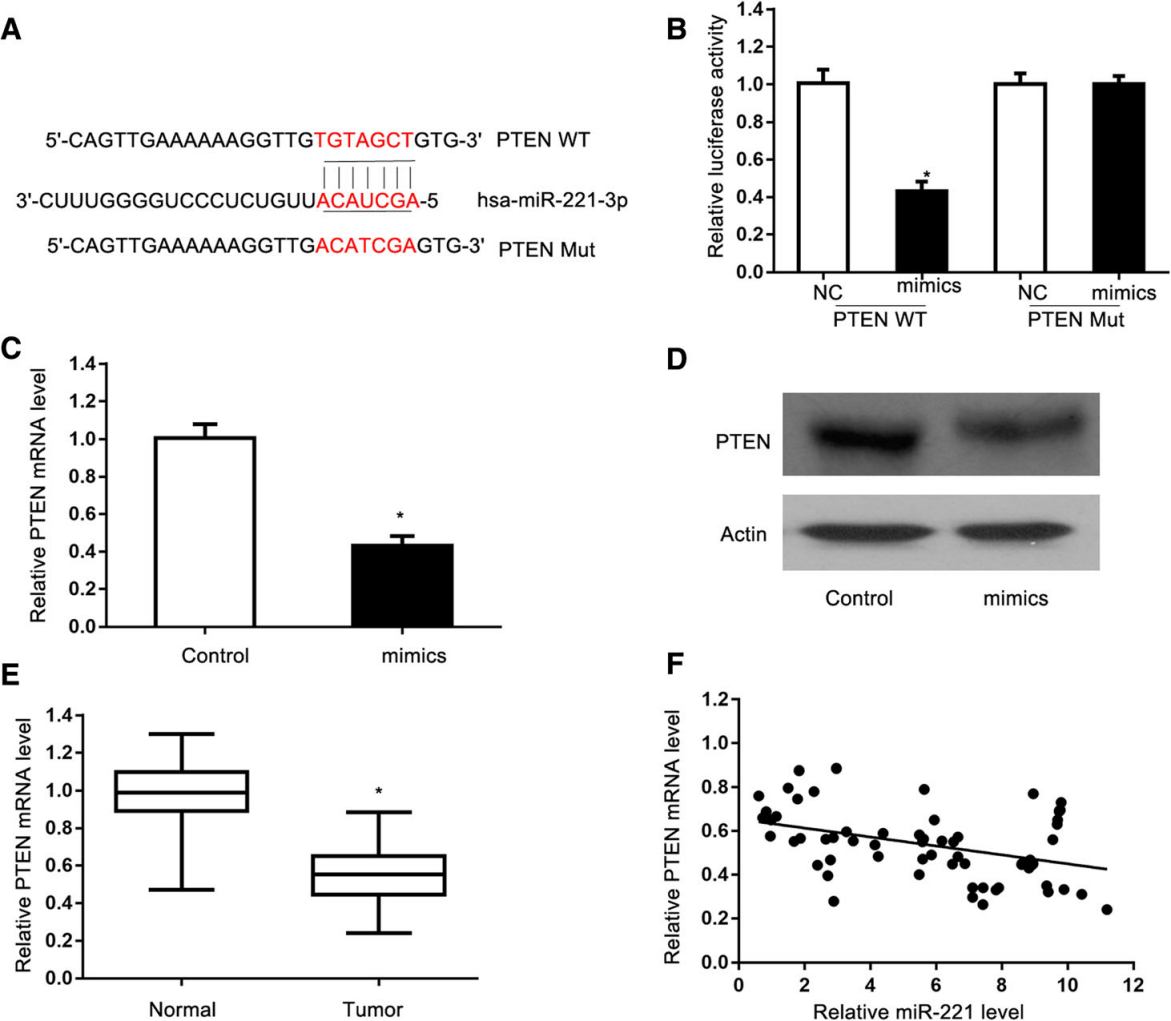
PTEN is a direct target of miR-221. **a** Binding sequences for miR-221 in the 3′-UTR of PTEN, and the mutations in the 3′-UTR of PTEN are presented. **b** Luciferase activity of the wild type PTEN 3′-UTR (Wt) and mutant T PTEN 3′-UTR (Mut) co-transfected with miR-221 mimics or a negative control (miR-NC) was measured. **c** RT-qPCR analysis of PTEN mRNA in A431 and SCC13 cells following transfection with miR-221 inhibitor or mimics. **d** Western blotting was used to detect PTEN protein expression in A431 and SCC13 cells following transfection with miR-221 inhibitor or mimics. **e** Relative PTEN mRNA expression levels were determined using RT-qPCR in CSCC tissues and adjacent non-tumorous gastric mucosae tissues. **f** Analysis of correlation between miR-221 and PTEN mRNA expression in CSCC tissues

We first used the TargetScan bioinformatics algorithm to explore the underlying mechanisms by which miR-221 exerts its function. PTEN was predicted as a potential target (Fig. 4a). Dual-luciferase reporter assay verified that miR-221 impaired the luciferase activity of the wild type PTEN 3′-UTR (WT) but not the MUT 3′-UTR of PTEN in cells (Fig. 4b). Gene expression analysis indicated that PTEN mRNA expression was decreased after transfection of miR-221a mimic in cells (Fig. 4c). Similar results were also achieved in Western blot analysis; miR-221 mimic decreased the PTEN level in cells (Fig. 4d). qRT-PCR analysis showed that PTEN mRNA expression levels were lower in CSCC tissues than adjacent non-tumorous tissues (Fig. 4e). Correlation analysis between miR-221 and PTEN mRNA expression in CSCC tissues demonstrated an inverse relationship. In all, miR-221 can directly target PTEN in CSCC cells (Fig. 4f).

### miR-221 regulates AKT signaling pathway

**Fig. 5:**
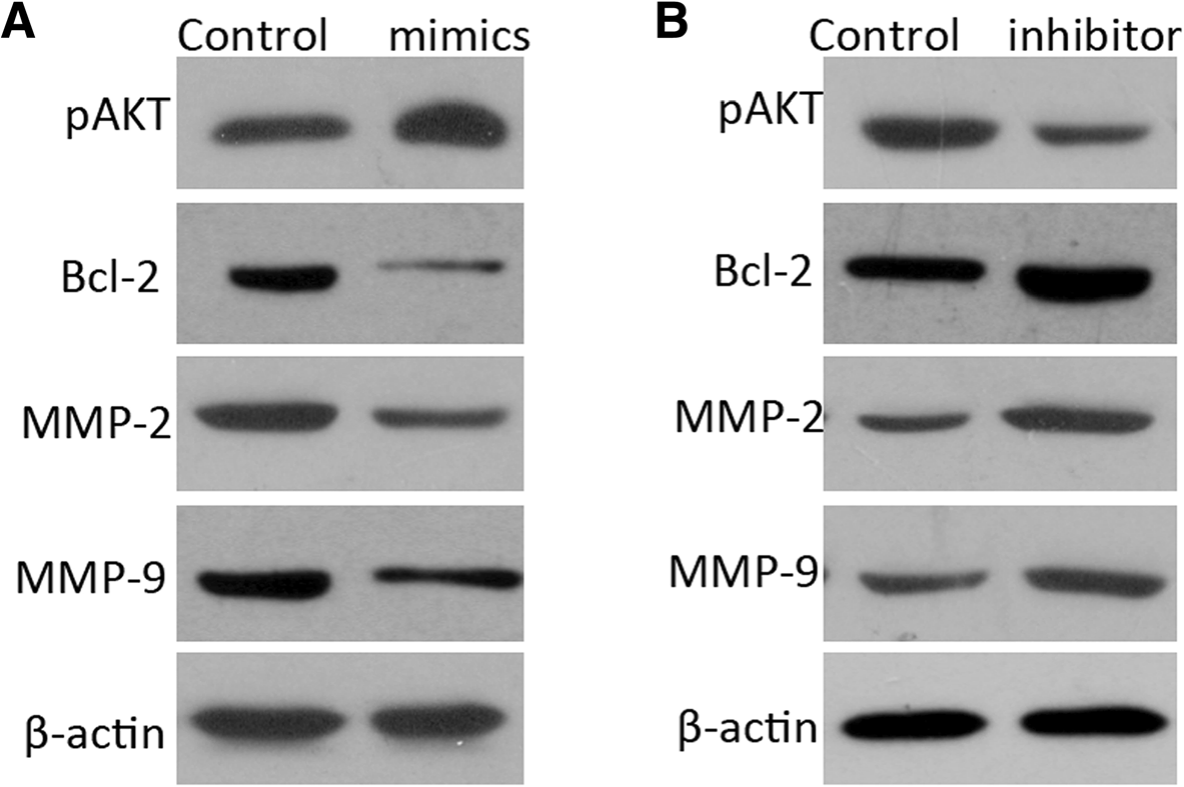
Impact of miR-221 in Akt pathway. **a**, **b** Western blot analysis of pAkt, cyclin D, Bcl-2, and MMP2/9 of cells following transfection with miR-221 inhibitor or mimics. β-actin was used as a negative control

We next explored whether the AKT signaling pathway was involved in miR-221 mediated cellular functions miR-221 in CSCC cells. Western blot analysis showed that transfection of cells with miR-221 mimic could enhance pAkt expression (Fig. 5a). In addition, the expression of Bcl-2, cyclin D, MMP2 and MMP9, all of which are regulated by pAkt, was slightly upregulated in the miR-221 mimic group (Fig. 5a). The opposite situation was found in cells transfected with miR-221 inhibitor (Fig. 5b).

## Discussion

In this study, we determined that miR-221 is increased in CSCC tissues and cell lines. We also found that miR-221 regulates several hallmarks of CSCC including cell growth and colony formation. Although the molecular mechanisms of miR-221 are elucidated in several types of cancer, the role of miR-221 in CSCC development still remained poorly understood. Therefore, it is of great value to understand the function of miR-221 in CSCC carcinogenesis.

miR-221 has been shown to serve tumor promoting roles in different types of human cancer. Ma et al. found that high expression of miR-221 in exosomes of the peripheral blood was positively associated with poor clinical prognosis of gastric cancer [17]. Li et al. demonstrated that up-regulation of miRNA-221 could target apoptotic protease activating factor-1, which further promotes ovarian cancer cell proliferation and indicates a poor prognosis [18]. Low miR-221-3p expression may lead to the poor prognosis of triple-negative breast cancer patients through regulating PARP1 [19]. However, there exist few reports concerning the potential function of miR-221 in human CSCC progression. In the current study, we elucidated the potential role of miR-221 in the malignant progression of CSCC.

We measured our collection of CSCC clinical samples, and observed that in tumor samples, the expression of miR-221 was higher than in normal tissues. Hence, we speculated that miR-221 may act as a tumor oncogene miRNA and its aberrant expression may be linked with advanced progression of human CSCC. Thus, we set the focal point on the functions and molecular mechanisms of miR-221 in human CSCC. At the cellular level, by transfecting cell lines with miR-221 mimics and miR-221 inhibitor, we found that miR-221 regulates the cell proliferation, colony formation and migration of CSCC cells, crucial steps in tumor progression. We further adopted bioinformatics analysis, TargetScan 6.2, to determine how miR-221 acts as an oncogene. Luciferase activity assay indicated direct targeting of PTEN by miR-221. In this study, we found that miR-221 can specifically target PTEN, and suppress PTEN protein expression.

PTEN protein is a classic tumor suppressor in various human cancers. The PTEN gene is located on chromosome 10q23.31 [20, 21]. PTEN functions as a negative regulator of the PI3K/Akt pathway through dephosphorylation of phosphatidylinositol 3,4,5 trisphosphate. PTEN is involved in regulation of cellular proliferation, apoptosis and metastasis during progression of cancers [22, 23]. Akt is a subfamily of the serine/threonine kinase family. It modulates the function of numerous substrates related to cell proliferation, apoptosis and invasion and is implicated in the progression of several tumors [24]. In our study, we observed that knockdown of miR-221 in CSCC cells leads to a decrease of the expression level of pAkt, and further influences the expression levels of other Akt-regulated proteins, such as Bcl-2, cyclin D1, and MMP2/9.

## Conclusions

In summary, our investigation provides effective evidence for the first time that miR-221 expression is upregulated in CSCC tissues and cells. Moreover, miR-221 promotes cell growth by targeting PTEN. These results provide strong evidence that miR-221 is implicated in the initiation and development of CSCC. All of these data hint that miR-221 may provide a potentially important therapeutic target for human CSCC.

## Abbreviations

CSCC: cutaneous squamous cell carcinoma
miRNA-221: microRNA-221
mRNAs: messenger RNAs
MUT: mutant type
PI: propidium iodide
SD: standard deviation
WT: wild type
